# pH-sensitive, polymer modified, plasma stable niosomes: promising carriers for anti-cancer drugs

**DOI:** 10.17179/excli2013-609

**Published:** 2015-01-06

**Authors:** Dena Tila, Seyede Narjes Yazdani-Arazi, Saeed Ghanbarzadeh, Sanam Arami, Zhaleh Pourmoazzen

**Affiliations:** 1 Tabriz University of Medical Sciences, Tabriz, Iran, Research Center for Pharmaceutical Nanotechnology; 2 Tabriz University of Medical Sciences, Tabriz, Iran, Department of Pharmaceutics, Faculty of Pharmacy; 3 Tabriz University of Medical Sciences, Tabriz, Iran, Student Research Committee, Faculty of Pharmacy; 4 Chemistry Department, Science Faculty, Azarbaijan Shahid Madani University,Tabriz, Iran

**Keywords:** pH-sensitive plasma stable niosome, cytotoxic effect, Mitoxantrone

## Abstract

The aim of this study was the design and evaluation of a novel plasma stable, pH-sensitive niosomal formulation of Mitoxantrone by a modified ethanol injection method. Cholesterol hemisuccinate was added instead of cholesterol in order to produce pH-sensitivity property and using PEG-Poly (monomethyl itaconate)-CholC6 (PEG-PMMI-CholC6) copolymer introduced simultaneously pH-sensitivity and plasma stability properties in prepared niosomes. The pH-sensitivity and cytotoxicity of Mitoxantrone niosomes were evaluated *in vitro* in phosphate buffer with different pHs as well as using human ovarian cancer cell line (OVCAR-3), human breast cancer cell line (MCF-7) and human umbilical vein endothelial cells (HUVEC). Results showed that both cholesterol derivatives bearing formulations had pH-sensitive property and were found to release their contents under mild acidic conditions rapidly. In addition, the PEG-PMMI-CholC6-based niosomes could reserve the pH-sensitivity after incubation in plasma. Both Mitoxantrone-loaded pH-sensitive niosomes showed higher cytotoxicity than the conventional niosomes on OVCAR-3 and MCF-7 cell lines. However, both pH-sensitive niosomes exhibited lower cytotoxic effect on HUVEC cell line. Plasma stable, pH-sensitive niosomes could improve the cytotoxic effect and reduce the side effects of anti-tumor drugs.

## Introduction

The ability to deliver drugs much more effectively and efficiently to the site of interest resulted in less harmful side effects and more valuable therapeutic actions, particularly in cancer treatment. High drug toxicity is a drawback for dose of chemotherapeutics, because side effects limit the drug dosage that can be administered (Hrushesky et al., 1992[18]; Wong et al., 2007[43]). Vesicles are promising carriers employed to deliver chemotherapeutic agents, antisense oligonucleotides and genes to various therapeutic targets, specifically (Chen et al., 2010[6]; Gabizon et al., 2004[12]; Mamot et al., 2003[27]; Petersen et al., 2012[31]). However, significant progress in overcoming many of the original obstacles for effective delivery of these agents has been limited. Different triggered release mechanisms have been designed for vesicles in order to promote drug concentrations in target tissues or cell compartments. These stimuli, including temperature light, enzymatic degradation and ultrasound have been efficiently used to initiate the breakdown of the bilayers (Akamatsu, 2009[1]; Cho et al., 2009[7]; Park et al., 2013[30]; Torchilin, 2009[39]; Zhou et al., 2012[48]; Zignani et al., 2000[49]). Many pH-sensitive systems are based on pH-responsive peptides or proteins that can efficiently trigger membrane fusion/disruption at acidic pH. Despite their interesting features, their clinical application may be limited due to potential immunogenicity. Another targeting strategy usually involves conjugation of vesicles with antibodies or other ligands. However, these methods have limitations due to the heterogeneous expression of the target receptors in diseased tissues. One of the common characteristics of diseased tissues (cancerous tumors, inflammation, infected areas, ischemia, arthritis, and atherosclerosis) is low pH in extracellular environment (Aojula et al., 2002[3]; Koren et al., 2012[21]; Negussie et al., 2010[28]; White et al., 1995[42]; Yuba et al., 2013[46]). The concept of pH-sensitive vesicles arises from the fact that certain enveloped viruses developed strategies to take advantage of the acidification of the endosomal lumen to infect cells, as well as from the observation in some pathological tissues. Recently, considerable efforts have been made to develop vesicles which are stable under normal physiological conditions but become destabilized and release their contents in acidic environment of tumor tissues, which is slightly acidic compared to healthy tissues. This enables the drug to be released selectively and specifically with greater efficiency from pH-sensitive vesicles in target sites and reduces side effects (Carafa et al., 2006[5]; Van den Bossche et al., 2011[40]; Yatvin et al., 1987[44]; Zhelev and Needham, 2002[47]).

Different classes of pH-sensitive vesicles have been proposed in the literature. The most commonly recognized concept involves the combination of phosphatidylethanolamine or its derivatives. In recent times, the use of novel pH-sensitive lipids, synthetic fusogenic peptides either encapsulated or incorporated in the lipid bilayer, and association of pH-sensitive polymers with vesicles, have been reported (Koutroumanis et al., 2013[22]; Sezaki et al., 1989[34]; Sulkowski et al., 2005[38]; Weiner, 1989[41]).

Recently the well modified polymers strategy was developed to improve targeting ability. Many polymers were investigated so far for the design of pH-sensitive vesicles based on poly(alkyl acrylic acid)s, succinylated PEG, biodegradable polyphosphazenes and N-isopropylacrylamide copolymers (Huynh et al., 2009[19]; Koutroumanis et al., 2013[22]; Luo and Jiang, 2012[26]; Salgado-Rodriguez et al., 2004[33]; Yessine et al., 2003[45]).

Although the pH-sensitive vesicles are very promising as a drug carrier, however, the lack of stability in blood and the short blood circulation time of pH-sensitive vesicles have remained as major drawbacks for their application *in vivo*. Recently, inclusion of polyethylene glycol (PEG)-derived lipids in vesicular bilayers has been shown that they are capable of inhibiting the rapid uptake of vesicles by the reticuloendothelial system which results in much longer *in vivo* circulation half-life. A main concern for pH-sensitive vesicles or any other polymer derivatives is the detrimental effect of PEGylation on the pH-sensitivity. Another issue regarding polymer-coated vesicles is their partial loss of pH-responsiveness following incubation in biological fluids. Protein adsorption on the pH-sensitive vehicles may stabilize the system and/or extract the copolymer from the lipid bilayers partially (Ducat et al., 2011[10]; Felber et al., 2012[11]; Ishida et al., 2006[20]; Løkling et al., 2003[25]; Sudimack et al., 2002[37]). 

Mitoxantrone (MTO) has been used extensively as a component of chemotherapeutic regimens for a number of fatal diseases, including leukemia, lymphoma, cancers of the breast and prostate, and to treat multiple sclerosis. However, MTO has been reported to provide only limited clinical benefit in intraperitoneal chemotherapy due to severe systemic toxicity, notably dose-related cardiotoxicity. Various delivery systems have been developed to reduce the dose-limiting systemic toxicities of MTO, including MTO-eluting beads and solid lipid nanoparticles. It has two physiologically dissociated groups (pKa values: 8.13 and 5.99) and it is soluble in water in spite of its lipophilicity (log P, 0.7) (Law et al., 1996[23]; Li et al., 1995[24]; Neuhaus et al., 2005[29]).

For decades, several vesicular systems have been studied as a potential system for delivering several different anticancer drugs. In this study we prepared plasma stable, pH-sensitive niosomes in order to improve cytotoxicity and selectivity of Mitoxantrone.

## Materials and Methods

### Materials

Mitoxantrone was obtained from Sigma-Aldrich Company (USA). Cholesterol (Chol) was obtained from Merck Company (Germany) and cholesterol hemisuccinate (CHEMS) was supplied from Sigma-Aldrich Company (USA). Cholesterol derivative (PEG-PMMI-CholC6) was synthesized in our group. Thiazolyl blue tetrazolium bromide (MTT), L-glutamine, penicillin, streptomycin, dimethyl sulfoxide and Sorenson buffer were obtained from Sigma-Aldrich (USA). RPMI-1640 medium and fetal bovine serum (FBS) were purchased from Gibco Company (USA). All solvents were HPLC grade and all other chemicals were analytical grade from Merck Company (Germany).

### Methods

#### Synthesis of cholesterol derivative

**Figure 1:**
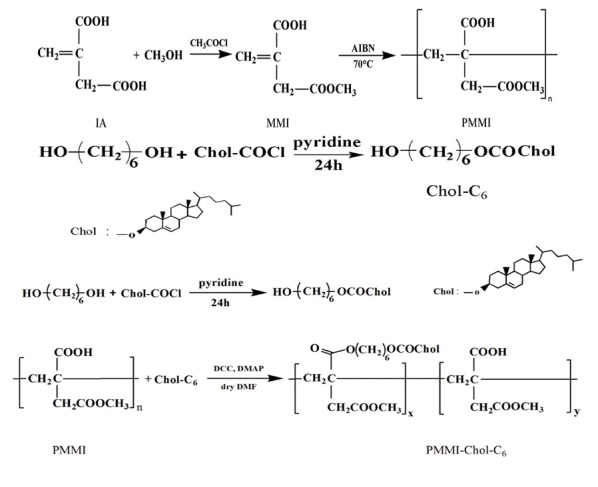
Synthesis pathway of PEG-PMMI-CholC6 copolymer

The PEG-PMMI-CholC6 copolymer was synthesized according to our previously published methods (Bagheri et al., 2013[4]). Synthesis pathway of PEG-PMMI-CholC6 copolymer was shown schematically in Figure 1(Fig. 1).

#### Niosome preparations

Niosomes were prepared by a method which was modified and described previously (Ghanbarzadeh et al., 2013[17]). Ingredients were dissolved in methanol/chloroform mixture in a 250 mL round-bottom flask and dried in a rotary evaporator under low pressure at 37 ºC to form a thin film on the flask. The Mitoxantrone aqueous solution (PBS pH 7.4) was added to the flask. Niosomes were formed by constant vortexing for 30 min and sonicated for 10 min in a bath sonicator. Prepared niosomes were subsequently extruded through polycarbonate filters (Millipore, USA) with a pore size of 100 nm using a handheld Mini Extruder (Avanti Polar Lipids Inc.).

#### Particle size and zeta potential measurement

Prepared niosomes were stored at 4 °C and ambient temperature for 3 months. Samples were taken on for analysis of particle size (PS) and zeta potential. For determination process, each sample was dispersed in PBS (pH 7.4) to a final volume of 10 mL and its particle size and zeta potential were analyzed using a Zetasizer (Malvern, UK).

#### Determination of the entrapped efficiency 

Dialysis tubes were employed for separation of un-entrapped drug during 24 hrs at 10 ºC. Residual drug content in niosomes was considered as encapsulated drug and encapsulation efficiency was measured after interruption of niosomes by methanol and measurement of the MTO quantity (Ghanbarzadeh and Arami, 2013[14]).

Encapsulation efficiency percent (EE%) was calculated using the following equation: EE (%) = [(C_total_–C_free_)/C_total_] ×100.

#### In vitro drug release

One milliliter of each niosomal formulation was placed in the dialysis tube containing polycarbonate membrane (cut-off: 100 nm) stirring (rpm=50) in a beaker containing 50 mL (37 °C) of phosphate buffer solution, and was adjusted to two pH values (pHs 6.5 and 7.4) (Ghanbarzadeh and Arami, 2013[13]). At predetermined time intervals, 2 mL of the dissolution medium was withdrawn from receiver compartment and drug content was measured spectrophotometrically (Ghanbarzadeh and Arami, 2013[13]). The same volume of fresh pre-warmed dissolution medium was replaced to keep the volume constant.

#### Physicochemical stability of niosomes

The stability of prepared niosomes was assessed in the case of particle size, zeta potential, encapsulation efficiency percent and remained drug at 4 °C and ambient temperature monthly over a period of three months.

#### Niosome plasma stability 

Particle size, drug leakage and stability of pH-sensitivity property of MTO-loaded niosome were evaluated after incubation in plasma (50:50 v:v) for 1 hr at 37 °C.

#### Cell lines

Cytotoxicity of prepared niosomes was assessed on the human breast cancer cell line (MCF-7), human ovarian cancer cell line (OVCAR-3) and on the human umbilical vein endothelial cells (HUVEC) (Obtained from Pasteur Inst. (Tehran, Iran). The cells were cultured in RPMI-1640 medium routinely, supplemented with 10 % fetal bovine serum, 100 U/mL penicillin G and 100 U/mL streptomycin, and incubated in 5 % CO_2_ at 37 °C subsequently. Subconfluent monolayer cells were detached from the culture T-75 flask by trypsin treatment and resuspended in the fresh RPMI-1640 medium. Cells were seeded at a density of 1×10^4^ cells per well in 96-well flat bottom plates with a final volume of 200 µL. 

#### Cytotoxicity study

To determine cytotoxicity activity of niosomal formulations, the cell viability assay, MTT (Thiazolyl blue tetrazolium bromide, a yellow tetrazole) assay, was employed. Briefly, MCF-7, OVCAR-3 and HUVEC cells were seeded in 96-well plate containing RPMI-1640 medium and subsequently incubated overnight. After this period, cells were treated with MTO-loaded conventional niosomes and two types of pH-sensitive niosomes at three concentrations (50, 100 and 200 µmol/L) for 72 hrs. Then, the incubation medium was replaced with 200 µL of fresh medium containing MTT (2 mg/mL). After incubation for extra 4 hrs at 37 °C, the culture medium containing MTT was removed and then 200 µL of DMSO: Sorencene buffer (8:1) was added to each well. Cell viability was detected by reading the absorbance of each well at 570 nm using a microplate reader after 15 min of shaking (Biotek, ELx 800, USA). 

#### Statistical analysis

Data analysis was performed using SPSS software (version 17). Analysis of variance (ANOVA) was employed for comparison of the difference among the groups. Data was expressed as Mean ± Standard Deviation and the P-value < 0.05 was considered as significance level. 

## Results and Discussion

### Preparation and characterization of niosomes

Table 1(Tab. 1) shows the composition of conventional and two types of pH-sensitive niosomes. These are optimized formulations which were obtained after our preliminary studies and experimental design. Immediately after preparation of niosomes, particle size, zeta potential and entrapment efficiency percent of three types of niosomes were investigated and presented in Table 2(Tab. 2). 

Mean particle sizes of conventional, CHEMS-based niosomes and PEG-PMMI-CholC6 bearing niosomes were 186.3 ± 26.3, 156.6 ± 16.3 and 168.3 ± 22.6 nm which did not show a significant difference among formulations. The particle size distribution was also showed a mono-modal distribution with low polydispersity index (data not shown). Encapsulation efficiency percent of prepared niosomes was fairly high and was found in the range of 70.9-73.2 % where there was not any significant difference among three noisome types. The zeta potential value of niosomal preparation is related to the stability of niosomes. Regarding the zeta potential of prepared niosomes, the zeta potential value of PEG-PMMI-CholC6 bearing pH-sensitive niosomes was significantly lower than pH-sensitive niosomes containing CHEMS and conventional niosomes, and zeta potential value slightly shifted towards neutral ( 12.1 ± 0.9 mV). The lower zeta potential may be attributed to the PEG layer formed on the surface of niosomes (Dadashzadeh et al., 2010[9]; Sadzuka and Hirota, 1997[32]; Shehata et al., 2008[35]).

### In vitro drug release study

**Figure 2:**
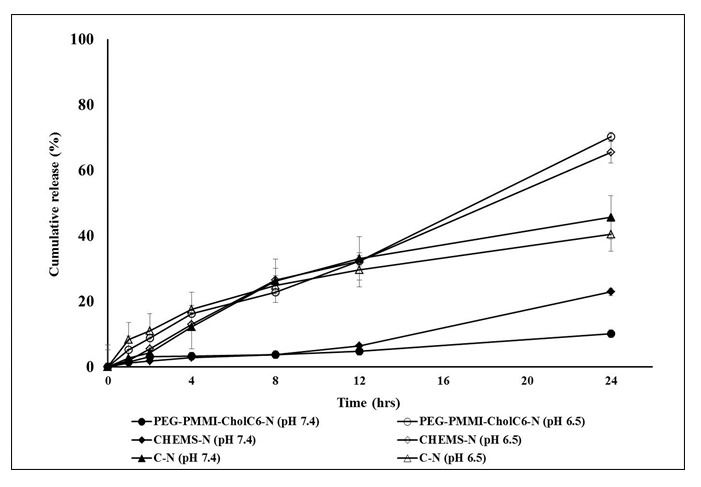
Cumulative drug release from different types of niosomes at pHs 6.5 and 7.4

To assay pH-sensitivity of prepared niosomes, *in vitro* release study was performed at two different pHs (7.4 and 6.5). Figure 2(Fig. 2) indicates that the cumulative drug release of conventional niosomes at pH 7.4 over 24 hrs (D24h) was significantly higher than the both types of pH-sensitive niosomes. In addition, the drug release rate from MTO-loaded PEG-PMMI-CholC6 bearing niosomes was significantly lower than CHEMS-based niosomes. On the other hand, at pH 6.5, both types of pH-sensitive niosomes released higher level of MTO over 24 hrs compared to pH 7.4, demonstrating suitable pH-sensitivity of these two types of niosomes. In the case of PEG-PMMI-CholC6 and CHEMS bearing niosomes, at pH 7.4, lower than 20 % and 10 % of the loaded MTO was released in 24 hrs, while at lower pH, a considerable increase was found in the release rate (65.4 and 70.2 %) indicating that these MTO-loaded PEG-PMMI-CholC6 and CHEMS-based niosomes may be suitable for intracellular drug delivery and drug delivery in cancerous tissues with mild acidic environment. These findings are in accordance with our preliminary investigation which showed that drug release rate from PEG-PMMI-CholC6 bearing micelles at pH 7.4 was slower than pH 6.5 (Ghanbarzadeh et al., 2013[15]). 

**Figure 3:**
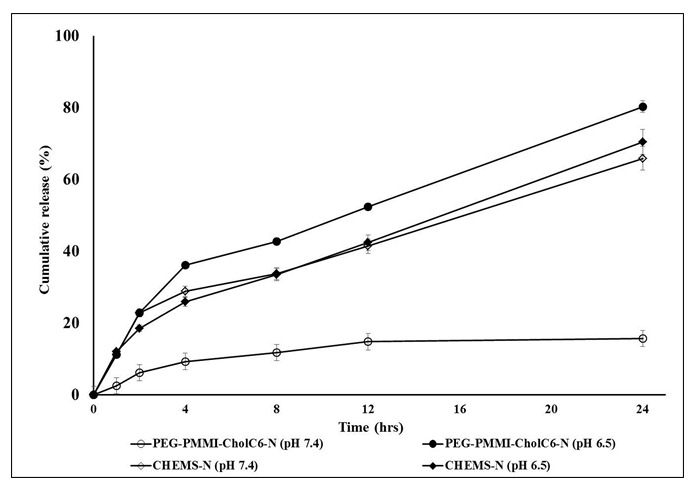
*In vitro* drug release of two types of pH-sensitive niosomes at pH 6.5 and 7.4 following 1 h incubation in human plasma

PH-sensitivity of prepared niosomes in plasma was assessed by *in vitro* release study of MTO from pH-sensitive niosomes in pHs 6.5 and 7.4 following 1 hr incubation in human plasma (50%, v/v) (Figure 3(Fig. 3)). The cumulative percent of released MTO from PEG-PMMI-CholC6 niosomes in pH 7.4 increased compared to non-incubated niosomes, however this increase was not significant (P>0.05). Conversely, CHEMS-based niosomes after 1 hr incubation in plasma lost their pH-sensitivity property fairly and released higher percent of MTO at pH 7.4 compared to those were not in contact with plasma. In contrast, as it was shown in Figure 3(Fig. 3), after incubation in plasma lowering the pH value to 6.5 caused an increase in the release rate of MTO from PEG-PMMI-CholC6 niosomes from 15.6 % at pH 7.4 to 80.2 % at pH 6.5 and this indicates the stability of pH-sensitive property of niosomes after being in contact with plasma proteins. The difference between released drug at two pHs, before and after incubation in plasma was not significant. However, after incubation in plasma, the cumulative percent of released drug over 24 hrs from CHEMS-based niosomes did not differ significantly in two pHs. 

These data indicated that pH-sensitive property of PEG-PMMI-CholC6 based niosomes would be preserved in the blood and these types of niosomes could be stable in neutral pH and release MTO just in the slight acidic environments, where often present in tumor tissue or in intracellular acidic endosomal compartment. While the CHEMS-based niosomes lasted this property and became unstable in plasma and released their cargo in the circulation. Therefore, in order to use *in vivo*, they should be protected in blood stream from opsonization and reticuloendothelial system. In this light, our previous unpublished study verified that using PEG-cholesterol instead of cholesterol could also preserve the pH-sensitivity property of niosomes after incubation in plasma. Therefore, plasma stable carriers can release the drugs at the target sites without wasting them during the circulation and can be promising carriers for intravenous delivery systems.

### Physicochemical stability study of niosomes 

Prepared niosomes were stored at 4 °C and 25 °C for a period of 3 months. The particle size distribution, zeta potential, entrapment efficiency percent and drug content of the MTO-loaded niosomes were analyzed at the end of each month and presented in Table 3(Tab. 3). 

Normally, vesicles are at risk of aggregation, fusion and leaking in ambient temperature. However, storage at refrigerator temperature minimizes the niosomes physical instability. Prepared niosomes were found to be generally stable in terms of aggregation or fusion, where particle size and zeta potential of niosomal formulation did not differ significantly after three months storage at 4 °C (P>0.05). On the other hand, storing at ambient temperature for three months resulted in an increase in particle size of all three types of niosomes (P<0.05). 

There was also no significant change in entrapment efficiency percent of the MTO niosomes after 3 months storage at 4 °C (P>0.05, Table 3). Mean initial EE% of three types of niosomes were 71.9 ± 2.3, 73.2 ± 1.6 and 70.9 ± 1.5 % and after three months storage at 25 °C, average EE% of MTO in niosomal formulations were found to be 58.1 ± 2.6, 59.4 ± 3.2 and 57.3 ± 2.6 %, respectively. Therefore, niosomes could be an effective carrier to minimize drug leakage from carrier at refrigerator temperature where higher percentage of drug was maintained in niosomal formulations compared to those stored at ambient temperature. This is predictable due to the fact that the higher temperatures resulted in higher fluidity of bilayers and as a result higher drug leakage (Angelini et al., 2011[2]; Cho et al., 2007[8]; Ghanbarzadeh et al., 2013[16]; Shimanouchi et al., 2009[36]).

To evaluate chemical stability of MTO niosomes, the drug content of niosomes was analyzed after three months storage at both refrigerator and ambient temperatures. Results revealed that MTO was relatively stable at niosomes stored at refrigerator conditions compared to room temperature. After three months, the mean MTO contents of niosomes were 87.1 % and 70.8 %, at 4 °C and 25 °C, respectively. Generally, physicochemical stability study results indicated that keeping the vesicular products in refrigerated condition minimizes the physicochemical instability problems of vesicles.

### Plasma stability 

Serum proteins can destabilize pH-sensitive vesicles and partially detach the copolymer from the lipid bilayers. Particle size and drug leakage of MTO-loaded PEG-PMMI-CholC6 niosomes were measured after one hour incubation in 50 % (v/v) human plasma. Results showed that incubation in plasma dose not significantly change niosomes size and encapsulated drug percent. The change of particle size and the drug loss or the leakage from niosomes were lower than 11 % and 30 %, demonstrated the stability of PEG-PMMI-CholC6-based niosomes in plasma which is considered to be due to the change of PEG on the surface of niosome. However, in CHEMS-based niosomes, incubation in plasma caused an increase in drug leakage and particle size growth, (P<0.05) (data not shown).

The plasma stability study and release profile of MTO-loaded PEG-PMMI-CholC6 niosomes, after incubation in plasma, indicated the stability of pH-sensitive property of MTO-loaded PEG-PMMI-CholC6 niosomes.

### Antiproliferative study 

**Figure 4:**
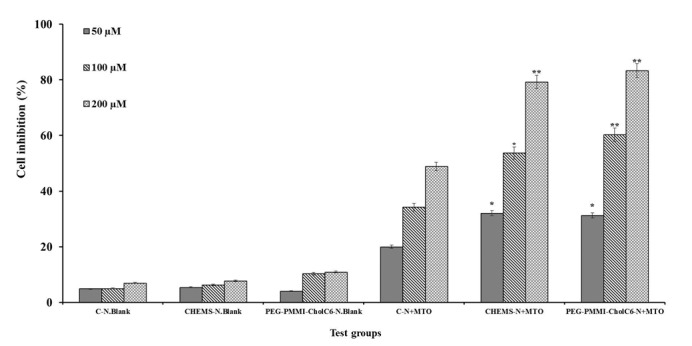
Cytotoxicity effect of MTO niosomes on MCF-7 cell line. Blank conventional niosomes (C-N.Blank), blank CHEMS niosomes (CHEMS-N.Blank), blank PEG-PMMI-CholC6 niosomes (PEG-PMMI-CholC6-N.Blank), MTO-loaded conventional niosome (C-N+MTO), MTO-loaded CHEMS niosomes (CHEMS-N+MTO) and MTO-loaded PEG-PMMI-CholC6 niosome (PEG-PMMI-CholC6-N+ MTO). (Data are the mean values of three replications, *P<0.05, **P<0.01 and ***P<0.001 compared with C-N+MTO).

**Figure 5:**
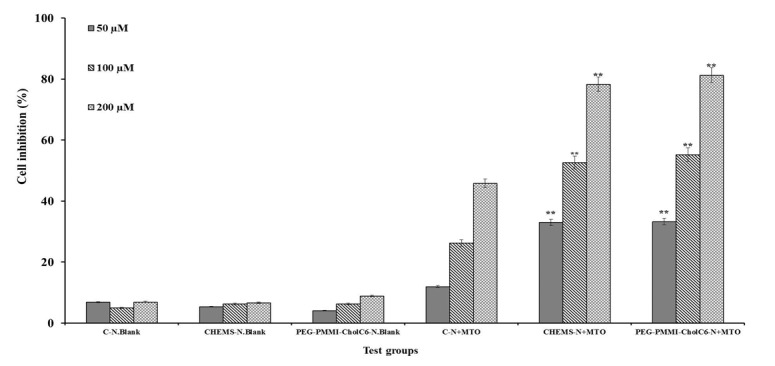
Cytotoxicity effect of MTO niosomes on OVCAR-3 cell line. Blank conventional niosomes (C-N.Blank), blank CHEMS niosomes (CHEMS-N.Blank), blank PEG-PMMI-CholC6 niosomes (PEG-PMMI-CholC6-N.Blank), MTO-loaded conventional niosome (C-N+MTO), MTO-loaded CHEMS niosomes (CHEMS-N+MTO) and MTO-loaded PEG-PMMI-CholC6 niosome (PEG-PMMI-CholC6-N+ MTO). (Data are the mean values of three replications, *P<0.05, **P<0.01 and ***P<0.001 compared with C-N+MTO).

**Figure 6:**
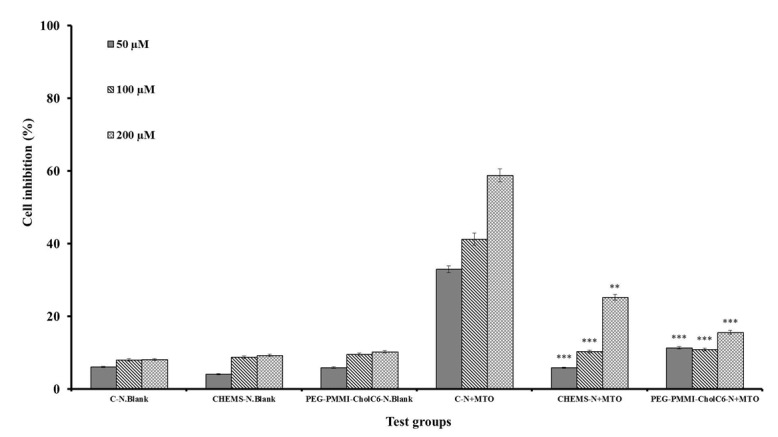
Cytotoxicity effect of MTO niosomes on HUVEC cell line. Blank conventional niosomes (C-N.Blank), blank CHEMS niosomes (CHEMS-N.Blank), blank PEG-PMMI-CholC6 niosomes (PEG-PMMI-CholC6-N.Blank), MTO-loaded conventional niosome (C-N+MTO), MTO-loaded CHEMS niosomes (CHEMS-N+MTO) and MTO-loaded PEG-PMMI-CholC6 niosome (PEG-PMMI-CholC6-N+ MTO). (Data are the mean values of three replications, *P<0.05,**P<0.01 and ***P<0.001 compared with C-N+MTO).

Cytotoxicity activity of conventional and both types of pH-sensitive niosomes with three concentrations were compared with corresponding blank niosomes on OVCAR-3, MCF-7 and HUVEC cell lines (Figures 4-6(Fig. 4)(Fig. 5)(Fig. 6), respectively). Cell viability was expressed as the ratio of the levels of formazan produced by treated cells to those produced by control (non-treated) cells. As it was shown, the control blank niosomes of each one did not display any significant cytotoxicity on any of cell lines (P>0.05) compared to non-treated groups. However, viability of OVCAR-3 and MCF-7 cells treated with both types of MTO-loaded pH-sensitive niosomes and conventional niosomes significantly decreased compared to respective blank niosomes. Non-viable OVCAR-3 cell count increased up to 33.83, 72.26 and 78.30 % in the case of conventional, CHEMS bearing pH-sensitive niosomes and PEG-PMMI-CholC6-based pH-sensitive niosomes containing 200 µmol MTO, respectively. Both types of pH-sensitive MTO niosomes had the higher antiproliferative activities (P<0.05) compared to conventional niosomes in each three concentrations.

In contrast, compared to both types of pH-sensitive niosomes, conventional niosomes displayed stronger cytotoxicity on HUVEC cells, as a healthy and control cell line, than OVCAR-3 and MCF-7 cell lines (P<0.05).

The results of MTT test confirmed the pH-sensitivity of both CHEMS and PEG-PMMI-CholC6 bearing niosomes, where these niosomes had higher cytotoxicity on cancer cell lines, compared to the normal cells. This property is advantageous due to the reduction of cytotoxicity of anti-cancer drugs on healthy cells and resulting side effects.

## Conclusions

The mechanism of many anticancer agents currently used currently for tumor treatment is based on cytotoxic effects. The non-target toxicity of chemotherapy is a major undesirable side effect that limits dose and therapeutic window. The method which is using polymers in order to modify the surfaces of vesicles has been studied as a way to increase the sensitivity and stability of vesicles. The addition of polymers can also exhibit *in vivo* prolonged circulation times and increase drug accumulation in tumors. In the present work, two types of pH-sensitive niosomes were prepared by using two cholesterol derivatives. Both niosomal formulations showed pH-sensitivity *in vitro*. Plasma stable niosomes were developed to improve the efficiency of the Mitoxantrone as antitumor drug and the effectiveness of the vesicles as a delivery system was studied. These niosomes were stable at physiological pH but they released their contents after being exposed to the acidic pH of the tumor tissues.

## Acknowledgements

The authors would like to thank the Research Center for Pharmaceutical Nanotechnology, Tabriz University of Medical Sciences, Tabriz, Iran. 

## Conflict of interest

The authors report no conflicts of interest.

## Figures and Tables

**Table 1:**
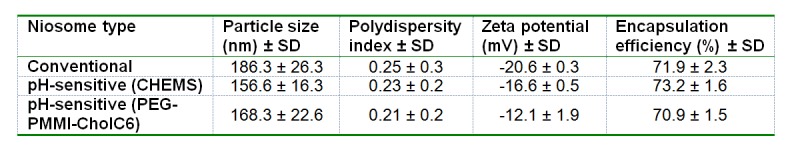
Composition of different types of prepared niosomes [Cholesterol (Chol), PEG-PMMI-CholC6, Tween 60 and cholesterol hemisuccinate (CHEMS) (µmol/mL)]

**Table 2:**
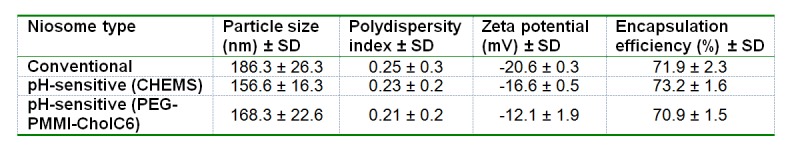
Characteristics of different type of niosomes

**Table 3:**
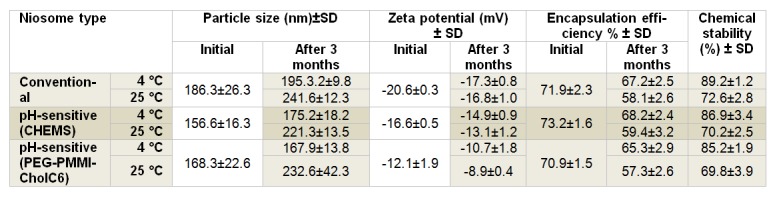
Stability of prepared vesicles during storage at 4°C and 25°C for three months

## References

[1] Akamatsu K (2009). Development of a thermal neutron-sensitive liposome for a novel drug delivery system aiming for radio-chemo-concurrent cancer therapy. Radiat Phys Chem.

[2] Angelini G, Chiarini M, De Maria P, Fontana A, Gasbarri C, Siani G (2011). Characterization of cationic liposomes. Influence of the bilayer composition on the kinetics of the liposome breakdown. Chem Phys Lipids.

[3] Aojula HS, Offerman S, Aojula RR, Hutchinson AP, Nicklin S, Clarke DJ (2002). Cloaking cytolytic peptides for liposome-based detection and potential drug delivery. Biochim Biophys Acta (BBA) - Biomembranes.

[4] Bagheri M, Pourmoazzen Z, Entezami AA (2013). Synthesis, characterization and liquid crystalline behavior of poly(monomethyl itaconate)s with new pendant cholesterol moieties. Iran Polym J.

[5] Carafa M, Di Marzio L, Marianecci C, Cinque B, Lucania G, Kajiwara K, Cifone MG (2006). Designing novel pH-sensitive non-phospholipid vesicle: Characterization and cell interaction. Eur J Pharmaceut Sci.

[6] Chen X, Wang X, Wang Y, Yang L, Hu J, Xiao W (2010). Improved tumor-targeting drug delivery and therapeutic efficacy by cationic liposome modified with truncated bFGF peptide. J Control Release.

[7] Cho EC, Lim HJ, Kim HJ, Son ED, Choi HJ, Park JH (2009). Role of pH-sensitive polymer–liposome complex in enhancing cellular uptake of biologically active drugs. Mat Sci Eng.

[8] Cho EC, Lim HJ, Shim J, Park JY, Dan N, Kim J (2007). Effect of polymer characteristics on structure of polymer–liposome complexes. J Colloid Interface Sci.

[9] Dadashzadeh S, Mirahmadi N, Babaei MH, Vali AM (2010). Peritoneal retention of liposomes: Effects of lipid composition, PEG coating and liposome charge. J Control Release.

[10] Ducat E, Deprez J, Gillet A, Noël A, Evrard B, Peulen O (2011). Nuclear delivery of a therapeutic peptide by long circulating pH-sensitive liposomes: Benefits over classical vesicles. Int J Pharm.

[11] Felber AE, Dufresne M-H, Leroux J-C (2012). pH-sensitive vesicles, polymeric micelles, and nanospheres prepared with polycarboxylates. Adv Drug Delivery Rev.

[12] Gabizon A, Shmeeda H, Horowitz AT, Zalipsky S (2004). Tumor cell targeting of liposome-entrapped drugs with phospholipid-anchored folic acid–PEG conjugates. Adv Drug Delivery Rev.

[13] Ghanbarzadeh S, Arami S (2013). Enhanced transdermal delivery of diclofenac sodium via conventional liposomes, ethosomes, and transfersomes. Biomed Res Int.

[14] Ghanbarzadeh S, Arami S (2013). Formulation and evaluation of piroxicam transferosomal gel: an approach for penetration enhancement. J Drug Delivery Sci Technol.

[15] Ghanbarzadeh S, Arami S, Pourmoazzen Z, Ghasemian-Yadegari J, Khorrami A (2013). Plasma stable, pH-sensitive fusogenic polymer-modified liposomes: A promising carrier for mitoxantrone. J Biomater Appl.

[16] Ghanbarzadeh S, Khorrami A, Pourmoazzen Z, Arami S (2013). Plasma stable, pH-sensitive non-ionic surfactant vesicles simultaneously enhance antiproliferative effect and selectivity of Sirolimus. Pharm Dev Technol.

[17] Ghanbarzadeh S, Valizadeh H, Zakeri-Milani P (2013). Application of response surface methodology in development of sirolimus liposomes prepared by thin film hydration technique. BioImpacts.

[18] Hrushesky WJM, Martynowicz M, Markiewicz M, von Roemeling R, Wood PA, Sánchez de la Peña S (1992). Chronotherapy of cancer: a major drug-deliver challenge. Adv Drug Delivery Rev.

[19] Huynh DP, Im GJ, Chae SY, Lee KC, Lee DS (2009). Controlled release of insulin from pH/temperature-sensitive injectable pentablock copolymer hydrogel. J Control Release.

[20] Ishida T, Okada Y, Kobayashi T, Kiwada H (2006). Development of pH-sensitive liposomes that efficiently retain encapsulated doxorubicin (DXR) in blood. Int J Pharm.

[21] Koren E, Apte A, Jani A, Torchilin VP (2012). Multifunctional PEGylated 2C5-immunoliposomes containing pH-sensitive bonds and TAT peptide for enhanced tumor cell internalization and cytotoxicity. J Control Release.

[22] Koutroumanis KP, Holdich RG, Georgiadou S (2013). Synthesis and micellization of a pH-sensitive diblock copolymer for drug delivery. Int J Pharm.

[23] Law SL, Ho CK, Jang TF, Chang P, Lin FM (1996). Anti-tumor effect of mitoxantrone-containing liposomes. Int J Pharm.

[24] Li SJ, Rodgers EH, Grant MH (1995). The activity of xenobiotic enzymes and the cytotoxicity of mitoxantrone in MCF 7 human breast cancer cells treated with inducing agents. Chem Biol Interact.

[25] Løkling K-E, Skurtveit R, Fossheim SL, Smistad G, Henriksen I, Klaveness J (2003). pH-sensitive paramagnetic liposomes for MRI: assessment of stability in blood. Magn Reson Imaging.

[26] Luo Z, Jiang J (2012). pH-sensitive drug loading/releasing in amphiphilic copolymer PAE–PEG: Integrating molecular dynamics and dissipative particle dynamics simulations. J Control Release.

[27] Mamot C, Drummond DC, Hong K, Kirpotin DB, Park JW (2003). Liposome-based approaches to overcome anticancer drug resistance. Drug Resist Upd.

[28] Negussie AH, Miller JL, Reddy G, Drake SK, Wood BJ, Dreher MR (2010). Synthesis and in vitro evaluation of cyclic NGR peptide targeted thermally sensitive liposome. J Control Release.

[29] Neuhaus O, Wiendl H, Kieseier BC, Archelos JJ, Hemmer B, Stüve O (2005). Multiple sclerosis: Mitoxantrone promotes differential effects on immuno-competent cells in vitro. J Neuroimmunol.

[30] Park SM, Kim MS, Park S-J, Park ES, Choi K-S, Kim Y-s (2013). Novel temperature-triggered liposome with high stability: Formulation, in vitro evaluation, and in vivo study combined with high-intensity focused ultrasound (HIFU). J Control Release.

[31] Petersen AL, Hansen AE, Gabizon A, Andresen TL (2012). Liposome imaging agents in personalized medicine. Adv Drug Delivery Rev.

[32] Sadzuka Y, Hirota S (1997). Physical properties and tissue distribution of adriamycin encapsulated in polyethyleneglycol-coated liposomes. Adv Drug Delivery Rev.

[33] Salgado-Rodríguez R, Licea-Claveríe A, Arndt KF (2004). Random copolymers of N-isopropylacrylamide and methacrylic acid monomers with hydrophobic spacers: pH-tunable temperature sensitive materials. Eur Polymer J.

[34] Sezaki H, Takakura Y, Hashida M (1989). Soluble macromolecular carriers for the delivery of antitumour drugs. Adv Drug Delivery Rev.

[35] Shehata T, Ogawara K-i, Higaki K, Kimura T (2008). Prolongation of residence time of liposome by surface-modification with mixture of hydrophilic polymers. Int J Pharm.

[36] Shimanouchi T, Ishii H, Yoshimoto N, Umakoshi H, Kuboi R (2009). Calcein permeation across hosphatidylcholine bilayer membrane: Effects of membrane fluidity, liposome size, and immobilization. Colloids Surf B Biointerfaces.

[37] Sudimack JJ, Guo W, Tjarks W, Lee RJ (2002). A novel pH-sensitive liposome formulation containing oleyl alcohol. Biochim Biophys Acta (BBA) - Biomembranes.

[38] Sułkowski WW, Pentak D, Nowak K, Sułkowska A (2005). The influence of temperature, cholesterol content and pH on liposome stability. J Mol Struct.

[39] Torchilin V (2009). Multifunctional and stimuli-sensitive pharmaceutical nanocarriers. Eur J Pharm Biopharm.

[40] Van den Bossche J, Al-Jamal WT, Yilmazer A, Bizzarri E, Tian B, Kostarelos K (2011). Intracellular trafficking and gene expression of pH-sensitive, artificially enveloped adenoviruses in vitro and in vivo. Biomaterials.

[41] Weiner AL (1989). Liposomes as carriers for polypeptides. Adv Drug Delivery Rev.

[42] White WI, Cassatt DR, Madsen J, Burke SJ, Woods RM, Wassef NM (1995). Antibody and cytotoxic T-lymphocyte responses to a single liposome-associated peptide antigen. Vaccine.

[43] Wong HL, Bendayan R, Rauth AM, Li Y, Wu XY (2007). Chemotherapy with anticancer drugs encapsulated in solid lipid nanoparticles. Adv Drug Delivery Rev.

[44] Yatvin MB, Tegmo-Larsson LM, Dennis WH, Ralph Green KJW (1987). Temperature- and pH-sensitive liposomes for drug targeting. Methods in enzymology.

[45] Yessine M-A, Lafleur M, Meier C, Petereit H-U, Leroux J-C (2003). Characterization of the membrane-destabilizing properties of different pH-sensitive methacrylic acid copolymers. Biochim Biophys Acta (BBA) - Biomembranes.

[46] Yuba E, Harada A, Sakanishi Y, Watarai S, Kono K (2013). A liposome-based antigen delivery system using pH-sensitive fusogenic polymers for cancer immunotherapy. Biomaterials.

[47] Zhelev DV, Needham D, Simon SA, McInotosh TJ (2002). Interactions of pH-sensitive peptides and polymers with lipid bilayers: Binding and membrane stability. Peptide-lipid interactions.

[48] Zhou W, An X, Wang J, Shen W, Chen Z, Wang X (2012). Characteristics, phase behavior and control release for copolymer–liposome with both pH and temperature sensitivities. Colloids Surf A: Physicochem Eng Aspects.

[49] Zignani M, Drummond DC, Meyer O, Hong K, Leroux J-C (2000). In vitro characterization of a novel polymeric-based pH-sensitive liposome system. Biochim Biophys Acta (BBA) - Biomembranes.

